# ATG5 and ATG7 Expression Levels Are Reduced in Cutaneous Melanoma and Regulated by NRF1

**DOI:** 10.3389/fonc.2021.721624

**Published:** 2021-08-12

**Authors:** Živa Frangež, Deborah Gérard, Zhaoyue He, Marios Gavriil, Yuniel Fernández-Marrero, S. Morteza Seyed Jafari, Robert E. Hunger, Philippe Lucarelli, Shida Yousefi, Thomas Sauter, Lasse Sinkkonen, Hans-Uwe Simon

**Affiliations:** ^1^ Institute of Pharmacology, University of Bern, Bern, Switzerland; ^2^ Department of Life Sciences and Medicine, University of Luxembourg, Belvaux, Luxembourg; ^3^ Biological Sciences Platform, Sunnybrook Research Institute, Sunnybrook Health Science Centre, Toronto, ON, Canada; ^4^ Department of Dermatology, Inselspital, Bern University Hospital, University of Bern, Bern, Switzerland; ^5^ Institute of Biochemistry, Medical School Brandenburg, Neuruppin, Germany; ^6^ Department of Clinical Immunology and Allergology, Sechenov University, Moscow, Russia; ^7^ Laboratory of Molecular Immunology, Institute of Fundamental Medicine and Biology, Kazan Federal University, Kazan, Russia

**Keywords:** autophagy, ATG5, ATG7, melanoma, NRF1, transcription factor

## Abstract

Autophagy is a highly conserved cellular process in which intracellular proteins and organelles are sequestered and degraded after the fusion of double-membrane vesicles known as autophagosomes with lysosomes. The process of autophagy is dependent on autophagy-related (ATG) proteins. The role of autophagy in cancer is very complex and still elusive. We investigated the expression of ATG proteins in benign nevi, primary and metastatic melanoma tissues using customized tissue microarrays (TMA). Results from immunohistochemistry show that the expression of ATG5 and ATG7 is significantly reduced in melanoma tissues compared to benign nevi. This reduction correlated with changes in the expression of autophagic activity markers, suggesting decreased basal levels of autophagy in primary and metastatic melanomas. Furthermore, the analysis of survival data of melanoma patients revealed an association between reduced ATG5 and ATG7 levels with an unfavourable clinical outcome. Currently, the mechanisms regulating ATG expression levels in human melanoma remains unknown. Using bioinformatic predictions of transcription factor (TF) binding motifs in accessible chromatin of primary melanocytes, we identified new TFs involved in the regulation of core ATGs. We then show that nuclear respiratory factor 1 (NRF1) stimulates the production of mRNA and protein as well as the promoter activity of *ATG5* and *ATG7*. Moreover, NRF1 deficiency increased *in vitro* migration of melanoma cells. Our results support the concept that reduced autophagic activity contributes to melanoma development and progression, and identifies NRF1 as a novel TF involved in the regulation of both *ATG5* and *ATG7* genes.

## Introduction

Melanoma is one of the most aggressive and treatment-resistant cancers worldwide. Despite new and promising therapeutic strategies, such as targeted therapies using BRAF and MEK inhibitors or novel immunotherapeutics, the prognosis of metastatic melanoma patients remains poor and is often associated with high tumor relapse rates. Additionally, current prognostic factors used in the clinics are not sufficient to identify all high-risk patients. Therefore, it is of increasing importance to develop new early-stage prognostic and diagnostic markers which help identify the patients at high risk of developing a more aggressive metastatic phenotype (1).

Autophagy is a highly dynamic process that is tightly regulated by so-called autophagy-related (ATG) proteins. Autophagy is crucial for the maintenance of cellular homeostasis and thus, it is no surprise that dysregulation of the autophagic process plays a crucial role in several diseases, including cancer. After years of research, the role of autophagy in cancer is still elusive and seems to be controversial, since depending on the stage and type of cancer it can have pro- or anti-tumorigenic properties (2). One of the main roles of autophagy as a tumor-supressing mechanism is maintaining genome integrity. Through its recycling procedure it removes defective proteins and organelles, such as defective mitochondria, and therefore prevents DNA damage and chromosomal instability, two attributes linked to cancer cell adaptation, enabling tumor progression and resistance to therapy (2). Two decades ago, the occurrence of a monoallelic deletion of Beclin-1 was identified in 40 to 75% of ovarian, breast and prostate cancers (3). A few years later, Qu and colleagues observed that monoallelic loss of Beclin-1 in mice correlates with high occurrence of tumors, such as hepatocellular carcinoma, lung adenocarcinoma and B cell lymphoma (4).

On the other hand, it has been shown that cancer cells activate autophagy in order to sustain sufficient nutrient supply due to their high metabolic demands (5). In addition, autophagy has been reported to support tumor cell resistance to anticancer therapy (6). We have recently shown that several proteins involved in the autophagic process; ATG5, Beclin-1 and BIF-1 are reduced in melanoma patients and that reduction of ATG5 and BIF-1 is associated with a less favourable patients’ outcome (7, 8). The exact mechanism regulating autophagy and its related proteins in melanoma, however, remains unknown.

Recently, researchers have started to uncover the broad network of transcription factors (TF) involved in regulation of autophagy. TF, such as transcription factor EB (TFEB), cyclic AMP-responsive element-binding protein 1 (CREB1) or Forkhead box O (FOXO) family of proteins, regulate the expression of numerous genes involved in autophagy initiation, autophagosome membrane elongation, substrate capture and autophagosome trafficking and their fusion with lysosomes (9–11). Interestingly, it has been demonstrated that changes in the activity or expression of only one TF could be enough to either suppress or induce autophagy (12).

Here we investigated the expression and transcriptional regulation of some of the core ATG proteins. We found that ATG5 and ATG7 are downregulated in primary and metastatic melanomas as compared to benign nevi and that patients with high levels of ATG5 and ATG7 in their tumors exhibited a better overall survival. Moreover, consistent with the reduced levels of ATG5 and ATG7, we found changes in the expression of autophagic activity markers, indicating reduced autophagy in melanomas as compared to benign nevi. Using bioinformatic predictions, we identified NRF1 as a new potential TF involved in the regulation of ATG5 and ATG7 and demonstrated that knockdown of NRF1 had an impact on protein and mRNA levels as well as promoter activity of both *ATG5* and *ATG7* genes. Furthermore, we found that knockdown of NRF1 increases the migration potential of melanoma cells. Our results emphasize the significance of autophagy for melanoma development and progression, and, for the first time, we provide data on the expression of ATG7 in melanoma. Taken together, we identified NRF1 as a novel TF involved in the regulation of ATG5 and ATG7 in melanoma and therefore provide novel insights into the transcriptional regulation of autophagy.

## Materials and Methods

### Study Design and Patients

This cohort study, aimed at investigating the role of autophagy in the pathogenesis of cutaneous melanoma, included archived tissue samples of consecutive melanocytic nevi, primary and metastatic melanomas obtained between the years 2003 and 2015 from patients at the Department of Dermatology, Inselspital, Bern, Switzerland. The tissue microarrays (TMA) containing tissue samples from benign nevi, primary and metastatic melanomas were constructed by the Department of Pathology, University of Bern. In total 98 benign nevi, 72 primary melanoma and 52 metastatic melanoma tissues were quantified. The study was approved by the Ethics Committee of the Canton of Bern.

### Immunohistochemistry

Paraffin-embedded tissue sections were deparaffinized and rehydrated with graded ethanol dilutions, after which antigen retrieval was carried out. Immunohistochemical staining was performed with the Dako REAL Detection System, using the Alkaline Phosphatase/RED kit according to the manufacturer’s instructions (Agilent Technologies, Santa Clara, California, USA). Staining was performed with monoclonal mouse anti-ATG5 antibody (11C3, Nanotools, 1:100 dilution, Teningen, DE), polyclonal rabbit anti-ATG7 antibody (Santa Cruz, 1:200 dilution, Dallas, Texas, USA), polyclonal anti-ATG16L1 (Lifespan Biosciences, 1:500 dilution, Washington, USA), polyclonal anti-p62 (Sigma-Aldrich, 1:200 dilution, Missouri, USA), polyclonal rabbit anti-LC3B antibody (Abgent, 1:100 dilution), and polyclonal anti-NRF1 (GeneTex, 1:50 dilution, California, USA). The staining intensities of these proteins were quantified as IODs using Image Pro Plus software as previously described (13, 14) or as mean OD using QuPath software as previously described (7, 15).

### Cells and Cell Culture

SK-MEL-5 human melanoma cell lines were cultured in RPMI 1640 + GlutaMAX medium supplemented with 10% fetal bovine serum, penicillin (100 U/mL), and streptomycin (100 mg/mL) at 5% CO_2_ at 37°C (all media were from Life Technologies, Carlsbad, California, USA).

### Immunoblot Analysis

Cell pellets from cultured cells were collected and lysed with the appropriate amount of lysis buffer (50 mM Tris [pH 7.4], 150 mM NaCl, 10% Glycerol, 1% Triton X-100, 2 mM EDTA, 10 mM NaPyrophosphate, 50 mM NaF, 200 μM Na_3_VO_4_). Lysis buffer was prepared freshly by adding 100 μM phenylmethylsulfonyl and a protease inhibitor cocktail (Sigma-Aldrich). The protein concentration was measured with the BCA Protein Assay kit (ThermoFisher Scientific, Waltham, Massachusetts, USA). 50 μg of cell lysate were separated on 12% SDS-PAGE gels and transferred to a polyvinylidene diuoride membrane (PVDF; Immobilon-P membrane, Merck Millipore, Burlington, Massachusetts, USA). After blocking in 5% milk in TBST (0.1% Tween-20 in 0.20 M Tris, 1.50 M NaCl [pH=7.6]) membranes were incubated with primary antibodies overnight at 4°C. Primary antibodies were purchased as follows: monoclonal anti-ATG5 antibody (11C3, 1:1000 dilution, Nanotools), monoclonal anti-ATG7 antibody (D12B11, 1:1000 dilution, Cell Signalling, Massachusetts, USA), monoclonal anti-Beclin-1 antibody (D40C5, 1:1000 dilution, Cell Signalling), polyclonal anti-NRF1 (1:500 dilution, GeneTex), polyclonal anti-NFE2L2 (1:1000 dilution, GeneTex), polyclonal anti-p62 (1:1000 dilution, Cell Signaling), polyclonal anti-LC3B (1:1000 dilution, Novus Biologicals, Colorado, USA), and monoclonal anti-alpha*-*tubulin (clone B*-*5*-*1*-*2, 1:5000 dilution, Sigma-Aldrich, Missouri, USA). The membranes were washed three times with TBST and incubated with horseradish peroxidase (HRP)-conjugated secondary antibody (1:10000 dilution, GE Healthcare, Illinois, USA) in blocking buffer for 1 h at room temperature. For the detection of HRP, chemiluminescent substrate (ECL-Plus Western Blotting Substrate, ThermoFisher Scientific) was used. Immunoblot signals were acquired on an Odyssey-Fc (LI-COR Biosciences, Nebraska, USA) and analysed using Image studio lite (LI-COR Biosciences).

### Quantitative Real-Time PCR

RNA was isolated with the ZR RNA MicroPrep kit (Zymo Research, Irvine, California, USA). After reverse transcription of isolated RNA to complementary DNA (cDNA), duplicate real-time PCRs were performed with the iQ SYBR Green Supermix (Bio-Rad Laboratories AG, California, USA) using the CFX Connect real-time PCR detection system (Bio-Rad Laboratories) or the Absolute Blue qPCR SYBR Green Low ROX Mix (ThermoFisher Scientific) using the Applied Biosystems 7500 Fast Real-Time PCR System, with 100 ng extracted genomic DNA, and 200 nM of each primer in 10 μL final PCR mix. The cycling variables were 3 min at 95°C followed by 40 cycles of 10 s at 95°C and 30 s at 55°C. Human primers were as follows: human ATG5, 5’-CAC AAG CAA CTC TGG ATG GGA TTG-3’ (forward) and 5’-GCA GCC ACA GGA CGA AAC AG-3’ (reverse); human ATG7, 5’-TCG AAA GCC ATG ATG TCG TCT T-3’ (forward) and 5’-CCA AAG CAG CAT TGA TGA CCA-3’ (reverse); human Beclin-1, 5’-CCA GGC GAA ACC AGG AGA GAC-3’ (forward) and 5’-GGG ATG AAT CTG CGA GAG ACA CC-3’ (reverse); human HPRT, 5’-TGG ACA GGA CTG AAC GTC TT-3’ (forward) and 5’-GAG CAC ACA GAG GGC TAC AA-3’ (reverse); human NRF1, 5’-AGC AAG CTA TTG TCC TCT GTA TCT CA-3’ (forward) and 5’-TGA GAG GCG GCA GTT CTG A-3’ (reverse); human NFE2L2, 5’- GAC GGA AAG AGT ATG AGC TGG AA-3’ (forward) and 5’-TGC GCC AAA AGC TGC AT-3’ (reverse). Primers were synthesized by Microsynth AG (Balgach, DE).

### siRNA Transfection

SK-MEL-5 cells were transfected with Lipofectamine 2000 (Invitrogen, California, USA) according to manufacturer’s instructions using 50 nM of gene-specific siRNAs against human NRF1 (siNRF1) (Dharmacon M-017924-01-0005); NFE2L2 (siNFE2L2) (Dharmacon M-003755-02-0005) or 50 nM of a negative control siRNA duplexes (siControl) (Dharmacon, D-001206{14-05). Cells were collected 48 h after transfection and processed for RNA extraction and protein lysates. Nuclear factor erythroid 2-related factor 2 (NRF2), also known as nuclear factor erythroid-derived 2-like 2, is a transcription factor encoded by the *NFE2L2* gene. To avoid confusion with the similarly named Nuclear Respiratory Factor 1 (NRF1), NRF2 protein is called throughout the manuscript by its gene name NFE2L2.

### Dual-Luciferase Assay

SK-MEL-5 cells were co-transfected with the pGL4.15 firefly luciferase vector containing 1 kb regions upstream of transcription start site of ATG5, ATG7 or Beclin-1 promoter sequences, pGL4.74 Renilla luciferase vector as an internal control and siRNAs (SMARTpool, Dharmacon) targeting selected transcription factors using the lipofectamine method described above. 48 h after transfection, cells were lysed in the luciferase lysis buffer according to the manufacturer’s instructions (Dual-Glo Luciferase Assay System, Promega, Dübendorf, DE) and luminescence was measured using a GloMax reader (Promega). Luciferase activity was normalized to that of Renilla luciferase activity.

### Wound Healing Assay

SK-MEL-5 melanoma cells were transfected as described above and were grown to 80% confluence in 24-well plates, then further incubated overnight in RPMI 1640 medium without fetal calf serum. A scratch was made on the cell monolayer using a plastic P100 pipette tip. The cells were then washed with phosphate-buffered saline (PBS) three times, and further cultured in RPMI 1640 medium. Pictures were taken immediately after the scratch and after incubation for 24 h. The scratched area was measured using ImageJ. The scratched area after 24 h was normalized to the initial size at t = 0 h and presented as percentage of wound closure.

### Analysis of Public DNase-Seq Data

Analysis of public DNase-seq data from human primary melanocytes generated by the Roadmap Epigenomics Mapping Consortium (GSM774243, GSM774244, GSM1027307, GSM1027312) {Bernstein, 2010 #402} were used. Raw data was downloaded from the Sequence Read Archive (SRA) and mapped to the human reference genome GRCh38.p12 downloaded from Gencode using the PALEOMIX pipeline (16). The DNase-seq peaks were called with HOMER (17) for each sample and the overlap between the four peak sets were derived with the ChIPpeakAnno R package (18). Bigwig tracks were generated with deepTools2 (19) and visualized in the Integrative Genomics Viewer (IGV) (20). Public NRF1 ChIP-Seq data for HeLa S3, HepG2, K562 and SK-N-SH (GSM935636, GSM2534537, GSM2424260 and GSM1003630) from the ENCODE project (21) were downloaded from the Gene Expression Omnibus repository and visualized in IGV.

### TEPIC

Transcription factor affinities were computed within the overlap of all identified open-chromatin regions in the DNase-seq data using TEPIC Version 2.2 (22, 23). Predicted TF affinities to motifs at open chromatin regions of *ATG5* and *ATG7* promoters were used to select candidate TFs. The precision of gene-based scores computed by TEPIC with any accessible chromatin assay have been validated using ChIP-seq data sets (22, 23).

### GSE3189 Analysis

The raw gene-expression profile GSE3189 was downloaded from the public database Gene Expression Omnibus (GEO, http://www.ncbi.nlm.nih.gov/geo/). GSE3189 is based on the Agilent GPL96 platform ([HG-U133A] Affymetrix Human Genome U133A Array). GSE3189 contains 70 samples, of which 7 are normal skin samples, 18 are benign skin lesion samples, and 45 are melanoma samples. The present study investigated only the melanoma and benign nevi samples (24). The Pearson correlation between the expression levels of selected TFs and the ATGs were calculated, and only those displaying robust association coefficients were considered for further analysis and plotted in the heatmap (R^2^>0.5 or R^2^<0.5). The analysis was performed on the normalized GSE3189 dataset obtained from GEO. The tidyverse and corrr packages were used in R version 3.6.0.

### Statistical Analysis

Data from at least three independent experiments is presented as means ± SD using Prism Software v6 (GraphPad, La Jolla, California, USA). Significant values were represented according to the following convention: p ≤ 0.05*; p ≤ 0.01**; p ≤ 0.001***; p value > 0.05 was considered not significant. The follow-up data on 102 primary and metastatic melanoma patients were divided for analysis into two groups according to the mean expression of ATG5, ATG7, and ATG16L1 in their tumors. The mean expression of ATG5, ATG7 and ATG16L1 in primary and metastatic melanomas were 101700.9 and 83535.2, 22192.5 and 8703.7, 59600.1 and 46045.8, respectively. The “high” group included patients with ATG levels that were higher than the mean value of the whole population. The “low” group included patients with ATG levels that were lower than the mean value of the whole population. OS was defined as the time from random assignment to death from any cause. It was analysed with the log-rank test and plotted as Kaplan-Meier survival curves.

## Results

### Decreased Expression of ATG5 and ATG7 in Melanoma Is Associated With Reduced Overall Survival

**Figure 1:**
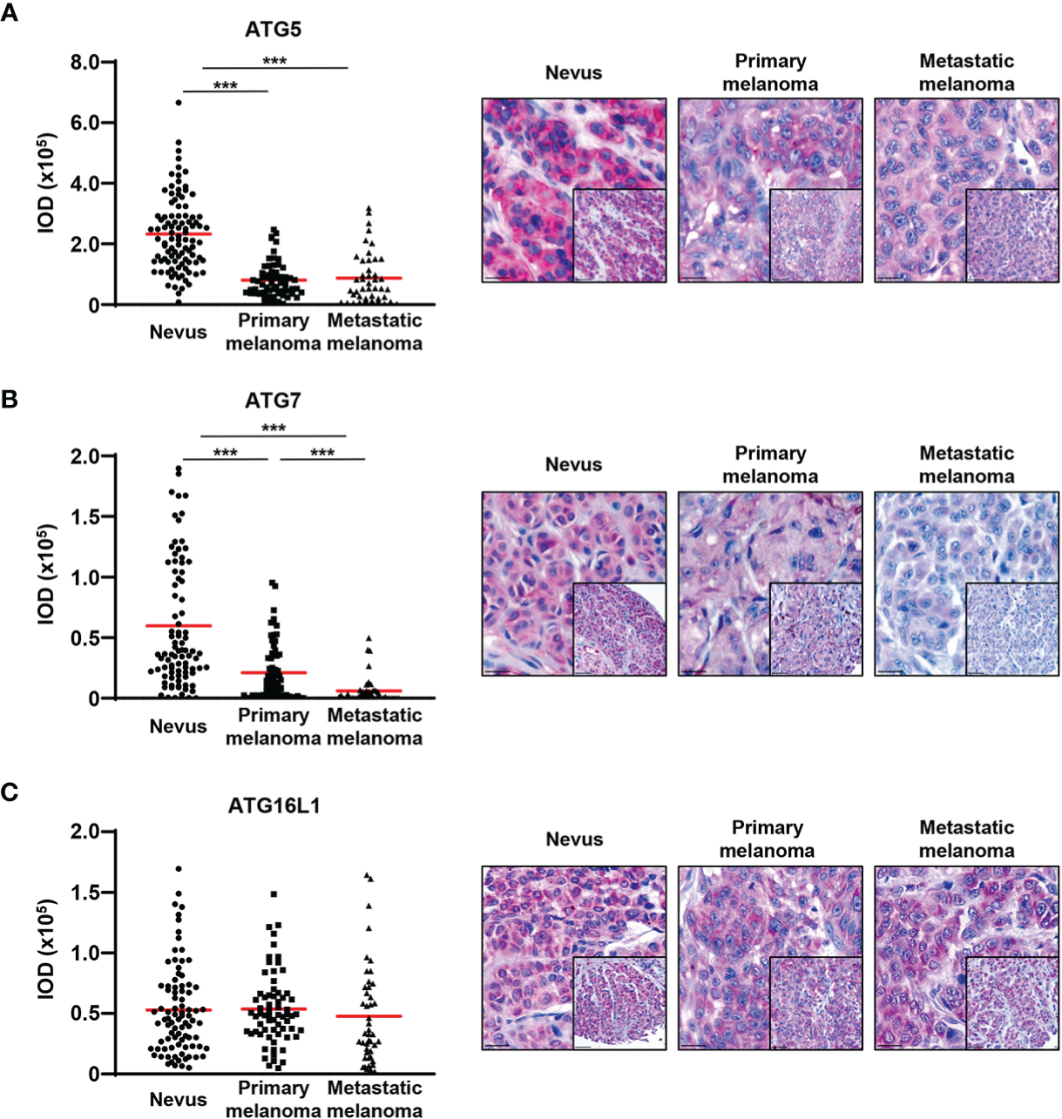
ATG5 and ATG7 levels are decreased in primary and metastatic melanomas compared with benign nevi. **(A–C)** Immunohistochemistry. Quantification of the ATG5, ATG7, and ATG16L1 signal intensity. Intensity (integrated optical density (IOD)) values for individual patients are presented. The red lines represent the mean of all values. Statistical differences were analysed by one-way ANOVA using a Kruskal-Wallis test and Dunn’s *post hoc* test (left panel). p ≤ 0.001***. Representative images of 98, 89 and 88 benign nevi as well as 67, 69 and 67 primary and 44, 44 and 45 metastatic melanomas for ATG5, ATG7, and ATG16L1, respectively (right panel). Scale bars 20 μm (inlets) and 50 μm.

In order to investigate the expression of ATG proteins and autophagic activity markers across several stages of melanoma biopsies, we constructed customized TMAs that contained tissue samples from benign nevi donors as well as from primary and metastatic melanoma patients. The expression levels of the autophagy proteins ATG5, ATG7, ATG16L1, and two autophagic markers, LC3B and p62, were assessed in melanoma patients by immunohistochemistry. In agreement with our previous results, we observed a significant decrease in ATG5 levels from benign nevi to primary melanomas. Interestingly, the levels of ATG5 were similarly decreased in both primary and metastatic melanomas (**Figure 1A**) (13). Next, we detected a consistent decrease in ATG7 expression from benign nevi to primary melanomas and in contrast to ATG5, the levels of ATG7 were further reduced in metastatic tissues (**Figure 1B**). On the other hand, we did not observe any differences in the expression of ATG16L1 between benign nevi, primary and metastatic melanoma specimens (**Figure 1C**).

**Figure 2:**
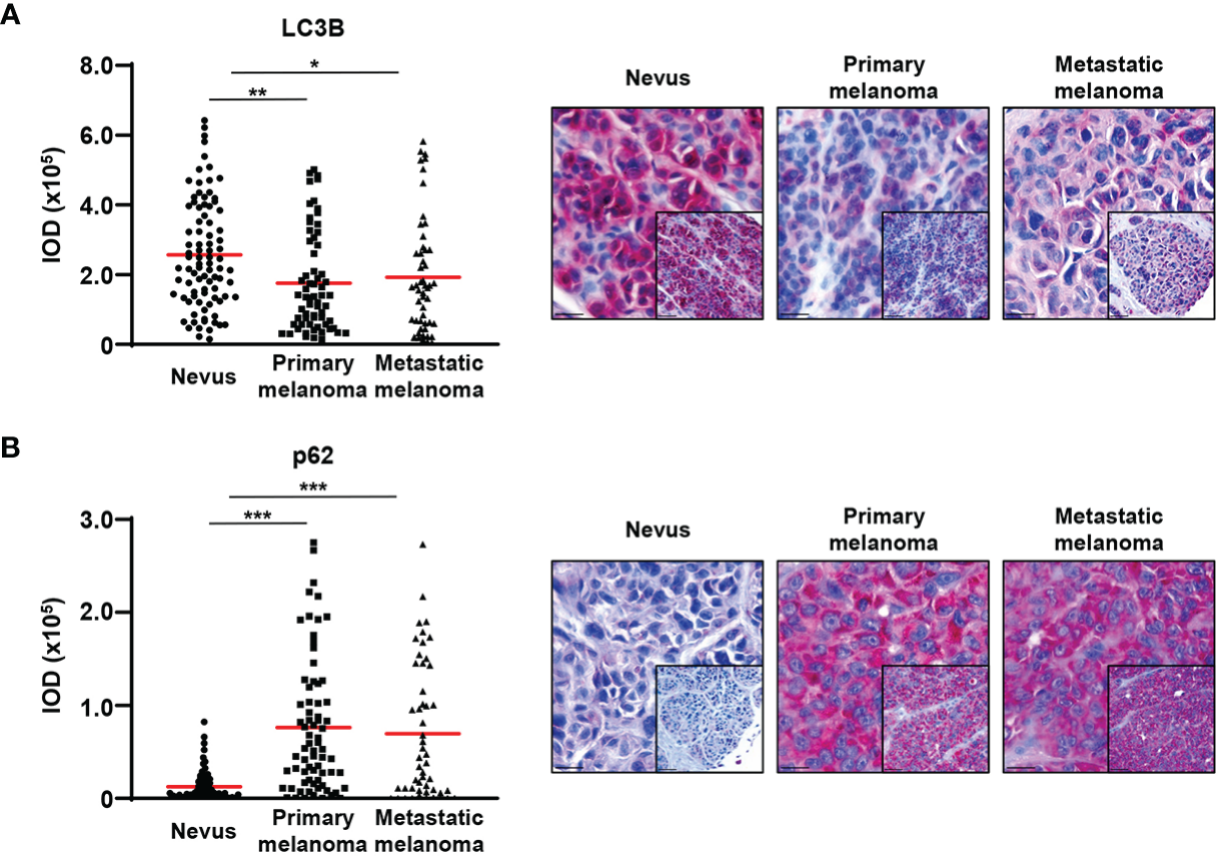
Decreased LC3B and increased p62 expression in primary and metastatic melanomas compared with benign nevi. **(A, B)** Immunohistochemistry. Quantification of the LC3B and p62 signal intensity. Intensity (integrated optical density (IOD)) values for individual patients are presented. The red lines represent the mean of all values. Statistical differences were analyzed by one-way ANOVA using a Kruskal-Wallis test and Dunn’s *post hoc* test (left panel). p ≤ 0.05*; p ≤ 0.01**; p ≤ 0.001***. Representative images of 88 and 75 benign nevi as well as 72 and 67 primary and 52 and 51 metastatic melanomas are shown for LC3B and p62, respectively (right panel).

To investigate whether the reduction of the expression of ATG5 and ATG7 results in reduced autophagy, we analysed *in vivo* levels of LC3B and p62. The lipidated form of LC3B is known to be bound to the inner and outer autophagosomal membrane and it has been shown that its levels correlate with the number of autophagosomes (25). To our knowledge, there is currently no specific antibody available that would identify the lipidated form of LC3B by immunohistochemistry. However, staining of the non-lipidated form is usually weak or not detectable (26, 27). Therefore, the LC3B staining largely reflects the amount of lipidated LC3B. We combined the LC3B data with an additional autophagic marker p62 and consistent with the expression of ATG5 and ATG7, we observed markedly lower LC3B and higher p62 levels in melanomas than in nevi, suggesting reduced autophagic activity in melanoma cells (**Figures 2A, B**). These data are in agreement with previously published work (13, 28).

**Figure 3:**
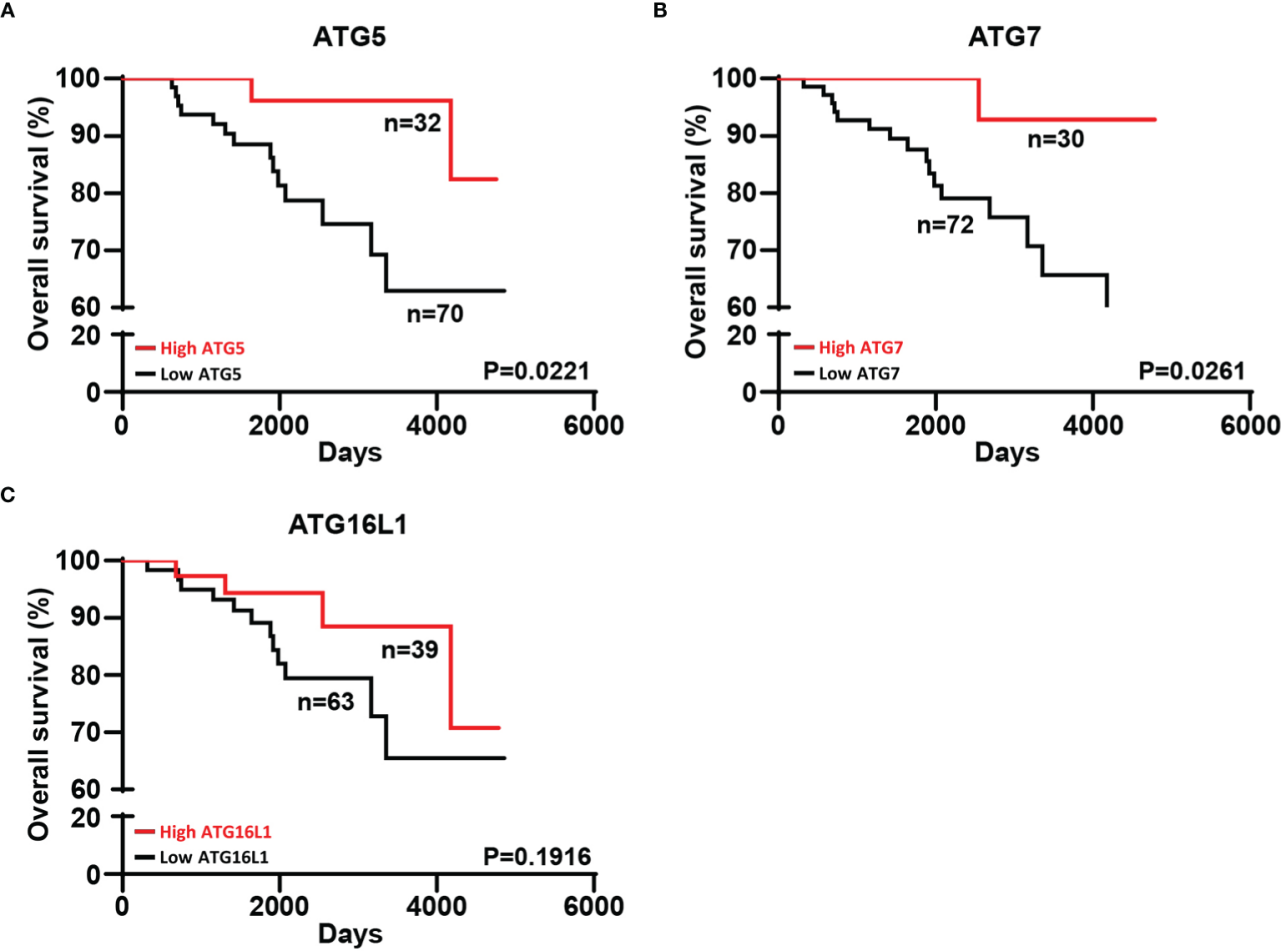
Melanoma patients with high expression of ATG5 and ATG7 in their tumors exhibited a better overall survival. **(A–C)** The same follow-up patients were divided into two groups (“high” and “low”) on the basis of the mean expression of ATG5, ATG7, and ATG16L1 in their tumors. Kaplan–Meier curves for overall survival are shown. Statistical analysis was performed using a log-rank test. The hazard ratios (HR) with their corresponding confidence intervals (95%CI) were estimated for each group. Strata \high ATG5” [HR=0.2136;95%CI (0.0786 to 0.5806)], strata \high ATG7” [HR=0.1423;95%CI (0.0507 to 0.3995)], strata \high ATG16L1” [HR=0.4801;95%CI (0.1773 to 1.300)].

Stratification of patients according to the mean expression levels of ATG5 and ATG7 in their tumors revealed a significantly improved overall survival (OS) in patients with elevated ATG5 and ATG7 levels (**Figures 3A, B**). Given the similar levels of ATG16L1 across all the different stages of melanoma progression, the expression of ATG16L1 was unrelated to the outcome of patients (**Figure 3C**).

Together with our previously published findings (8, 13) we show that the expression of autophagic proteins involved in the initiation and elongation phase of autophagy is reduced in melanoma, which is accompanied with a concomitant decrease of autophagy in these tumors. Moreover, our study, for the first time, associates the expression of ATG7 with melanoma development and progression. We demonstrate that the expression of ATG7 is reduced in primary melanoma and in contrast to ATG5, the expression of ATG7 is further reduced in metastatic melanoma tissues.

### NRF1 Is a Novel Transcription Factor Involved in Regulation of ATG5 and ATG7 Expression in Melanoma

**Figure 4:**
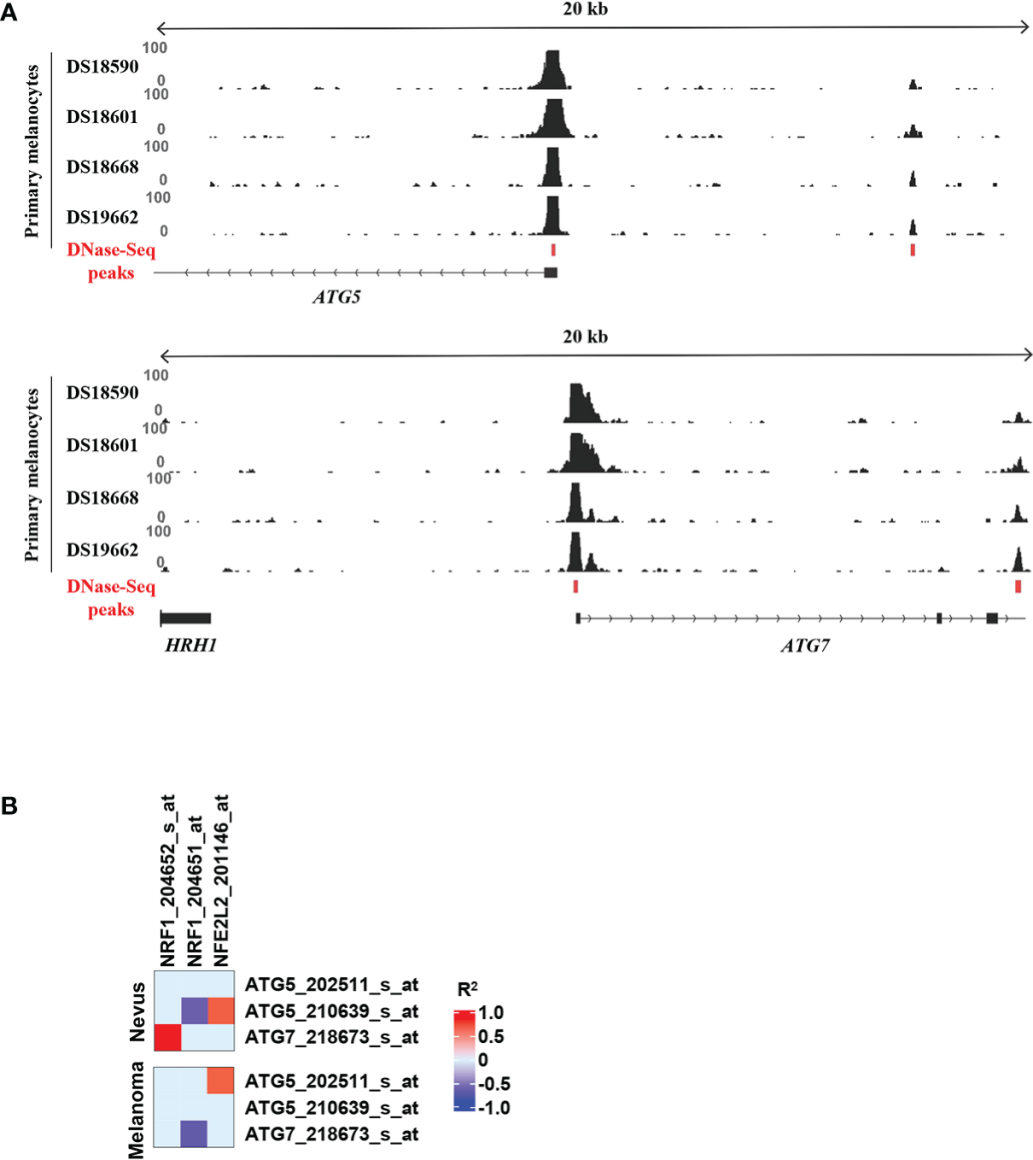
Selection of TFs with highest affinity scores for *ATG5* and *ATG7* promoters. **(A)** Prediction of binding of TFs to regulatory regions of *ATG5* and *ATG7* using the TEPIC tool. TF affinity scores were computed in the overlap of all DNase-seq identified open-chromatin peaks across the replicates in the proximity of the gene-transcription start site with TEPIC. DNase-seq data from all replicates are shown. **(B)** Heatmap of the GSE3189 gene-expression profile showing the correlation between NRF1 and NFE2L2 and *ATG5* and *ATG7*. R^2^ values higher than 0.5 are coded in red and values lower than -0.5 are coded in blue in the Figure continuous scale. Individual probe sets for each gene are listed.

Recent publications demonstrated the important role of TFs in the regulation of autophagy (9, 11, 12). We decided to search for novel TFs regulating autophagy, and specifically ATG5 and ATG7, in melanoma. Firstly, we set out to identify putative TFs that could regulate ATG5 and ATG7 in healthy melanocytes. To this end, we used existing DNase-seq data from primary melanocytes together with the bioinformatic analysis tool TEPIC to identify TF binding motifs located in accessible chromatin regions proximal to the transcription start sites (TSS) of the genes of interest (**Figure 4A**) (22, 23). The TF binding affinity calculations were performed for the promoter regions found to be reproducibly accessible across four biological replicates as shown in **Figure 4**.

Interestingly, only one of the top 10 motifs with highest affinity scores for *ATG5* and *ATG7* promoter regions was in common for both genes (**Tables S1**, **S2**). This motif corresponded to the binding site of nuclear factor 1 X-type (NFIX), a TF best known for its role in brain development but also implicated as tumor suppressor in various cancers (29). In addition, the top hits for *ATG5* included CREB and TFE family motifs that can be bound by CREB1 and TFEB, TFs known to be involved in regulation of ATG5 and several other autophagy-regulating genes (9–11). The TF with the highest affinity score for *ATG7* promoter region was the nuclear respiratory factor-1 (NRF1). Importantly, we found ChIP-seq data for genome-wide binding of NRF1 in HepG2, K562, SK-N-SH, and HeLa-S3 cancer cell lines in the data from the ENCODE project (https://www.encodeproject.org/). As predicted, we could see a clear enrichment for NRF1 binding at the TSS of *ATG7* (**Figure S1**). Although it was not found under the top 10 candidates for *ATG5* predicted by TEPIC, based on NRF1 ChIP-seq data, strong enrichment for NRF1 binding was also seen on the TSS of *ATG5* (**Figure S1**). Therefore, we decided to further investigate NRF1-mediated transcriptional regulation of *ATG7* and *ATG5* genes in melanoma cells. In addition to NRF1, we also included NFE2L2 in our investigation as an internal control TF. NFE2L2 has been shown to transcriptionally regulate numerous genes involved in autophagy, including *ATG5* and *ATG7*, and is therefore next to TFEB, CREB1 and FOXO3 one of the key regulators of autophagy (9, 10, 12, 30).

To investigate whether there is a connection between the expression of NRF1, NFE2L2 and ATGs, we analysed existing microarray data and investigated the gene expression profiles of 18 benign nevi and 45 primary melanoma tissue samples. We looked into the correlation between the expression of *ATG* genes and the genes of *NRF1* and *NFE2L2*. We found that expression levels of both TFs correlate with the studied *ATGs* in at least one of the tissue types (**Figure 4B**).

**Figure 5:**
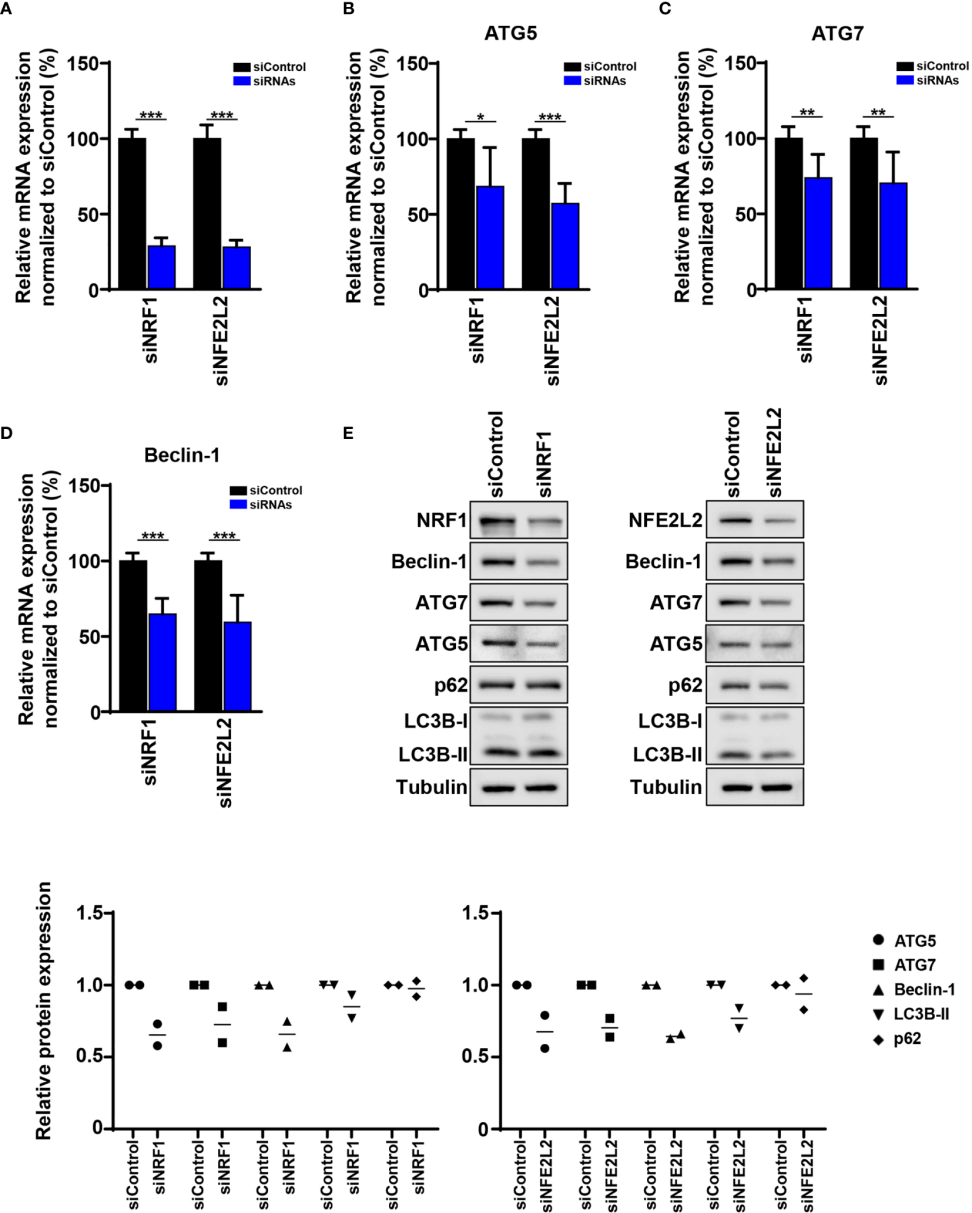
The mRNA and protein levels of ATG5, ATG7, and Beclin-1 are influenced by siRNA knockdown of *NRF1* and *NFE2L2*. **(A)** Quantitative polymerase chain reaction (qPCR). SK-MEL-5 melanoma cells were transfected with 50 nM siRNAs (Dharmacon) against *NRF1* and *NFE2L2* and Non-Targeting siRNA (Control). After 48 h *NRF1* and *NFE2L2* mRNA levels were determined by qPCR and normalized to *HPRT* mRNA levels. Statistical differences were analyzed by multiple t-test using the Holm-Sidak correction method (n=3). **(B–D)** Quantitative polymerase chain reaction (qPCR). SK-MEL-5 melanoma cells were transfected as described before and the mRNA levels of *ATG5*, *ATG7* and *Beclin-1* were determined by qPCR and normalized to *HPRT* mRNA levels. Statistical differences were analyzed by multiple t-test using the Holm-Sidak correction method (n=3). p ≤ 0.05*; p ≤ 0.01**; p ≤ 0.001***. **(E)** Immunoblot. SK-MEL-5 melanoma cells were transfected as described before. Expression of ATG5, ATG7, Beclin-1, LC3B, p62, NRF1, NFE2L2, and tubulin in siNRF1, siNFE2L2, and siControl cells. Lower panels: Quantification of the immunoblots. Values were normalized to the housekeeping protein and to siControl (n=2).

Since our previously published and current data show decreased expression of three of the core ATGs (ATG5, ATG7 and Beclin-1) in melanoma, we decided to study the impact of NRF1 and NFE2L2 on the expression of these ATGs. We performed a siRNA-mediated knockdown of *NRF1* and *NFE2L2* in melanoma cells (**Figure 5A**). Indeed, knockdown of *NRF1* and *NFE2L2* decreased the mRNA levels of *ATG5*, *ATG7* and *Beclin-1* (**Figures 5B–D**). Importantly, *NRF1* and *NFE2L2* knockdown led to a corresponding reduction in protein levels of ATG5, ATG7 and Beclin-1 (**Figure 5E**). In addition, knockdown of *NFE2L2* resulted in a slight decrease in LC3B-II levels, pointing to a possible reduction of autophagy (**Figure 5E**).

### NRF1 and NFE2L2 Regulate the Transcriptional Activity of *ATG5* and *ATG7*

**Figure 6:**
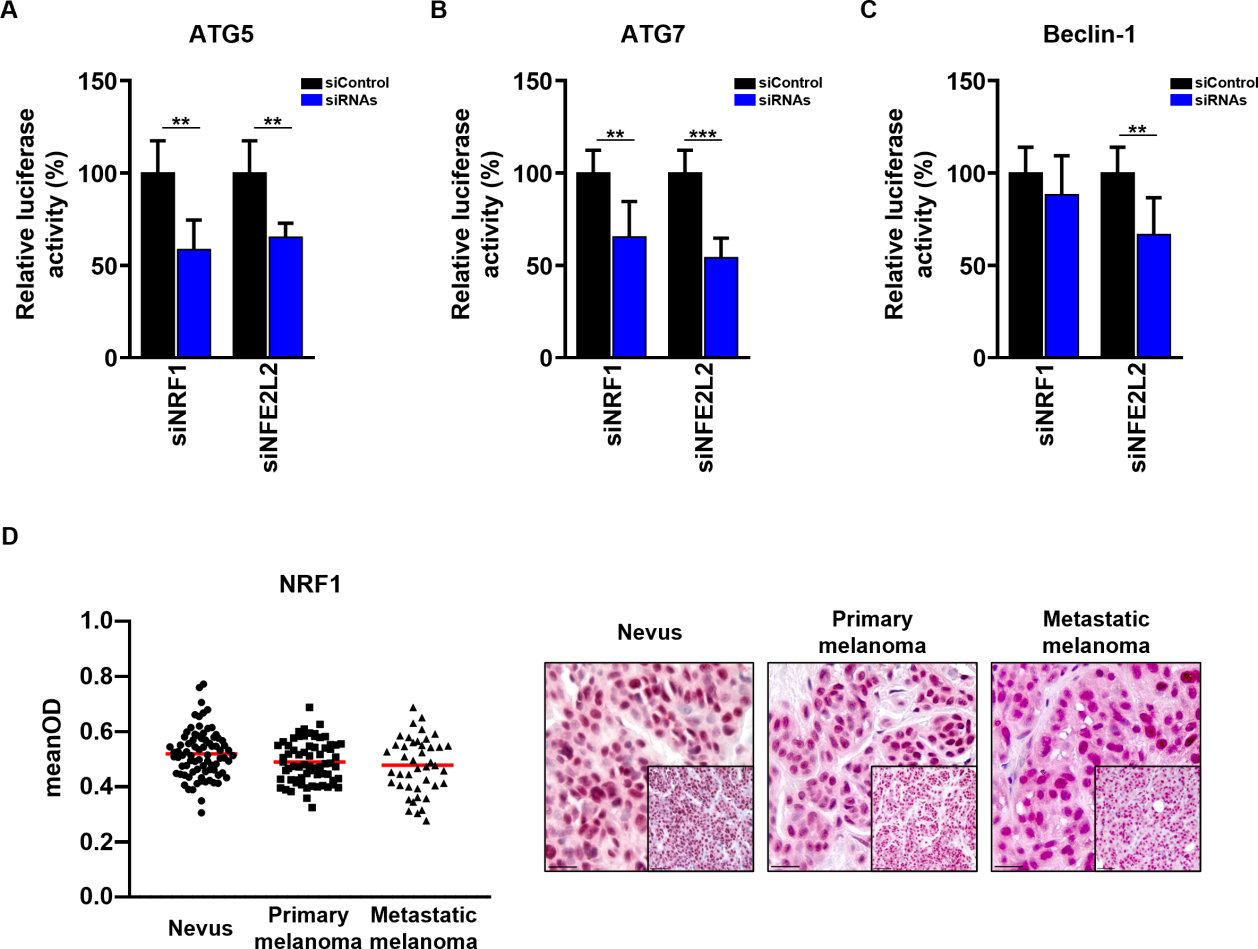
Validation of NRF1 and NFE2L2 regulation of ATG5, ATG7 and Beclin-1 promoter regions. **(A–C)** The Dual-Luciferase reporter plasmids (pGL4.74[hRluc/TK] and pGL4.15[luc2P/Hygro]) were co-transfected with 50 nM siRNAs (Dharmacon) into SK-MEL-5 melanoma cells. 48 h post transfection cells were assayed for luciferase activity. The firefly luciferase activities were normalized to renilla luciferase activity. The firefly luciferase activity of the cells transfected with siRNAs is represented as the percentage of activity relative to that of the cells transfected with Non-Targeting siRNA (siControl). Statistical differences were analyzed by multiple t-test using the Holm-Sidak correction method (n=3). p ≤ 0.01**; p ≤ 0.001***. **(D)** Immunohistochemistry. Quantification of the NRF1 signal intensity in the nucleus. Intensity (mean optical density (meanOD)) values for individual patients are presented. The red lines represent the mean of all values. Statistical differences were analyzed by one-way ANOVA using a Kruskal-Wallis test and Dunn’s *post hoc* test (left panel). Representative images of 80 benign nevi as well as 67 primary and 43 metastatic melanomas are shown (right panel). Scale bars 20 μm (inlets) and 50 μm.

In order to confirm that the selected TFs regulate promoter activity of the ATGs, we performed luciferase reporter assays, where we cloned *ATG5*, *ATG7* and *Beclin-1* minimal promoter elements (1 kb upstream from transcription start site) covering the most important TF binding sites into a firefly luciferase vector. We co-transfected melanoma cells with the firefly vector containing the promoters together with the siRNAs targeting TFs of interest. Interestingly, the luciferase assay showed that both TFs regulate several of the studied ATG promoters (**Figures 6A–C**). Specifically, we observed a decrease in promoter activity of *ATG5* and *ATG7* after both *NRF1* and *NFE2L2* knockdown, consistent with the results on protein and mRNA levels. Similarly, the knockdown of *NFE2L2* led to a decrease of Beclin-1 protein, mRNA and promoter activity; while the knockdown of *NRF1* decreased *Beclin-1* mRNA and protein expression, but did not have a direct impact on its promoter activity. This is in keeping with the investigated ENCODE ChIP-seq data that showed a clear enrichment for NRF1 binding at the promoters of *ATG5* and *ATG7*, but not at *Beclin-1* promoter (**Figure S1**). Potentially, NRF1 could regulate *Beclin-1* mRNA and protein expression by binding to distant regulatory regions such as enhancer regions that could not be captured in this experiment.

Interestingly, quantification of NRF1 immunohistochemistry staining in the same cohort of melanoma patients revealed no difference in the expression levels of NRF1 between benign nevi, primary and metastatic melanoma tissues (**Figure 6D**). Moreover, no correlation between ATG5 and ATG7 with NRF1 in benign nevi or melanoma tissues was observed (**Figure S2**). This phenomena could be explained by altered NRF1 activity in melanoma patients, impact of other TFs on *ATG* gene expression or the involvement of post-transcriptional regulation.

### NRF1 Knockdown Increases the Migration Potential of Melanoma Cells *In Vitro*

**Figure 7:**
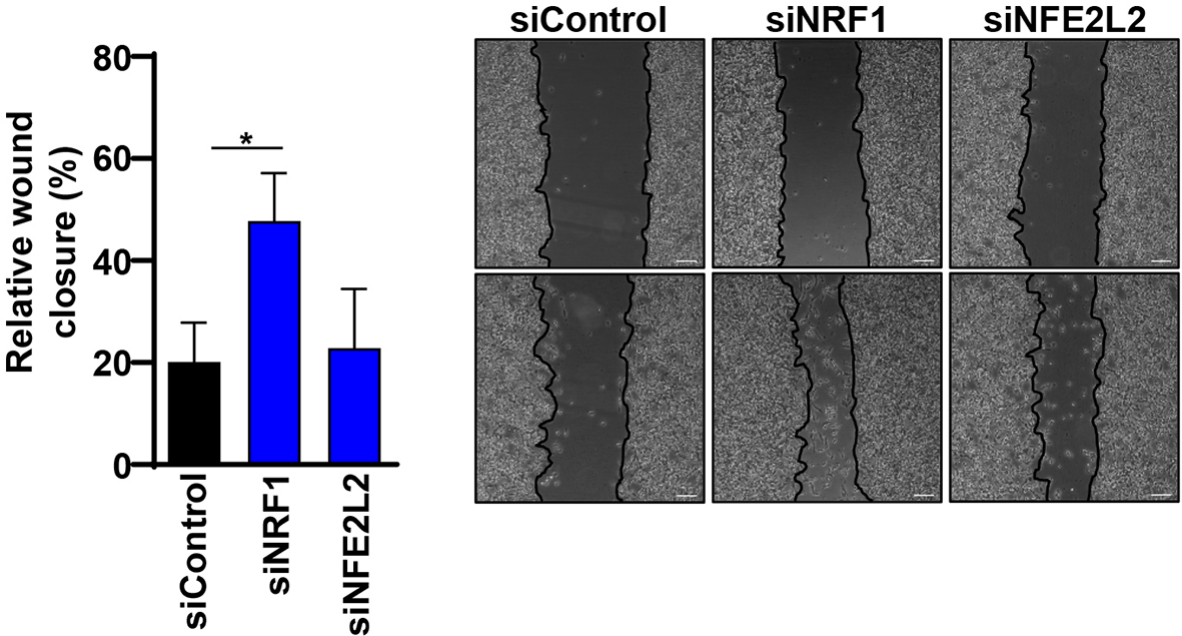
Knockdown of NRF1 increased the migration potential of melanoma cells. The wound-healing assay was made on overnight serum-starved, confluent melanoma cells by drawing a line across the bottom of the dish. The scratch was performed on cells that were previously treated with 50 nM siRNAs (Dharmacon) against NRF1 and NFE2L2 or Non-Targeting siRNA (siControl) for 48 h. Phase-contrast microscopy pictures were taken of the wounded area. Cell migration quantification of pictures is represented as the empty area from t=0 h normalized to empty area from t=24 h. Statistical analysis was performed by comparing all groups to the untreated control group using a one-way ANOVA, with Dunnett’s correction for multiple comparisons (n=3). p ≤ 0.05*. Scale 100 μm.

Since NRF1 and NFE2L2 have an impact on protein and mRNA levels of several ATGs and autophagy is known to play an important role in cancer, including influencing the epithelial-to-mesenchymal transition (EMT), we decided to evaluate the impact of NRF1 and NFE2L2 on the migration ability of melanoma cells (31). With this aim, we performed a wound healing assay on synchronized melanoma cells and compared the migration potential between specific siRNA-mediated knockdown of TFs and the non-targeting siRNA. Interestingly, knockdown of *NRF1* indeed increased the migration potential of melanoma cells, while NFE2L2 depletion had no effect (**Figure 7**).

## Discussion

Our previous studies have suggested that autophagy plays an important role in melanoma pathogenesis. In fact, ATG5 is downregulated in melanoma tissues and the reduced expression correlated with poor outcome for patients (13). Furthermore, lowering ATG5 expression promoted proliferation of melanoma cells by impeding oncogene-induced senescence (13). Moreover, Beclin-1, another core ATG protein involved in the initiation phase of autophagosome formation, was decreased in metastatic specimens compared to primary melanomas and benign nevi tissues (8).

In the current study, we observed a significant reduction in ATG5 and ATG7 expression in primary and metastatic melanomas compared to benign nevi. Furthermore, analyses of p62 and LC3B implied lower levels of autophagy in primary and metastatic melanomas when compared to benign nevi. Moreover, patients exhibiting higher levels of ATG5 and ATG7 in their tumors had a better overall survival. Similarly, other groups have recently investigated the expression of autophagic markers and ATG proteins in melanoma using immunostaining techniques. Mirraco et al. observed moderate to strong cytoplasmic staining and nuclear positivity of Beclin-1 in normal melanocytes, benign nevi, and the majority of dysplastic nevi. Moreover, they demonstrated a moderate LC3B staining of the cytoplasm in normal melanocytes and benign nevi as well as a heterogeneous LC3B expression pattern in melanomas and metastatic tissues (32). Furthermore, similarly to our results, a recent study showed increased p62 expression in primary and metastatic melanomas compared to benign nevi (28).

Since the expression levels of ATG5 and ATG7 are reduced in melanoma tissues and their expression levels correlated with the outcome of the patients, it is important to decipher the regulatory mechanisms responsible for the observed reduction. Currently, the details behind the regulation of the key autophagic proteins remain mostly unknown. Only recently scientists have started to understand the broad network of TFs involved in the regulation of autophagy. Among the mostly studied TFs are TFEB, a major regulator of lysosomal pathways, CREB1 and FOXO3 (9, 10, 12). Importantly, those studies showed that changes in expression or activity of a single TF can be sufficient to either induce or inhibit autophagy.

Using a novel bioinformatics tool called TEPIC, we identified putative TFs binding to promoter regions of *ATG5* and *ATG7*. Our main focus was on TFs showing the highest affinity either to ATG5 or ATG7 promoter. Elicited from our bioinformatics analysis, the top candidate for the *ATG7* promoter was NRF1. It should be noted that NFIX was found at both promoters with lower, but still relatively good affinity. Interestingly, ChIP-seq data with NRF1 in 4 different cancer cell lines depicted strong enrichment in close proximity of both *ATG7* and *ATG5* TSSs. In addition to NRF1, we also investigated NFE2L2 and used it as an internal control, since a recent study using MEFs showed that NFE2L2 deficiency resulted in decreased expression of several autophagy-regulating genes (30). NFE2L2 is a basic leucine zipper TF involved in the regulation of antioxidant genes. The role of NFE2L2 in cancer is still a matter of debate, since it has been reported to act pro- and anti-tumorigenic (33). Our top candidate NRF1 is one of the regulators of the cellular antioxidant defence system and activates the expression of some of the key genes that are required for respiration and mitochondrial DNA transcription and replication (34). An interesting finding was reported by Bhawe and Roy describing that NRF1 binding motifs are present in genes implicated in pathways regulating proliferation, invasion, and apoptosis, all major processes involved in carcinogenesis (35). In addition, a recent study showed that knockdown of NRF1 decreased the expression of ATG7 and overexpression of NRF1 increased protein levels of ATG7 in nucleus pulposus (NP) cells (36). Here we show that NRF1 indeed regulates the levels of ATG5, ATG7 and Beclin-1 at both mRNA and protein levels. In addition, by measuring the promoter activity of *ATG5*, *ATG7* and *Beclin-1*, we validated the transcriptional regulation of NRF1 at the *ATG5* and *ATG7* promoters. The NRF1 ChIP-seq data from the ENCODE project confirms the binding of NRF1 to *ATG5* and *ATG7* promoter regions in 4 different cancer cells (HepG2, K562, SK-N-SH, and HeLa-S3 cell lines). Moreover, consistently with the results from the gene reporter assays, no NRF1 binding was detected in the promoter region of Beclin-1. Since NRF1 regulates protein and mRNA levels of Beclin-1 but does not have an impact on its promoter activity, we assume that NRF1 could regulate the *Beclin-1* gene indirectly, by either binding to distal regulatory regions or by regulating the activity or expression of other TFs. In contrast to what we observed in patients samples, NRF1 knockdown did not influence autophagic activity *in vitro*. Perhaps knockdown of NFR1 and the subsequent decrease of ATG5 and ATG7 expression was not strong enough to have an impact on autophagy *in vitro*. Moreover, autophagy is a very complex process and there are several factors that could influence its levels in patients. It is unlikely that NRF1 is the sole driver of autophagy and melanoma progression in patients. In addition, knockdown of NRF1 could potentially result in an increase of other TFs that induce autophagy.

As assessed by immunohistochemistry, NRF1 expression did not show any difference between benign nevi, primary or metastatic melanoma tissues. One of the key regulators of NRF1 is peroxisome proliferator-activated receptor gamma coactivator 1-alpha (PPARGC1A or PGC1α), which has been shown to increase the *NRF1* gene expression and to coactivate the transcriptional function of NRF1 on promoters of genes such as *mitochondrial transcription factor A (mtTFA)*, which triggers transcription and replication of mtDNA (37, 38). Interestingly, elevated levels of PGC1α inversely correlates with invasive growth in melanomas and silencing of PGC1α increases the invasive potential of non-metastatic melanoma cells (39). Indeed, we observed that knockdown of NRF1 leads to an increased migration potential in melanoma cells. Moreover, PGC1α has been shown to regulate one of the key TFs involved in regulation of autophagy, TFEB, by occupying its promoter (40). Therefore, it is possible that decreased NRF1 activity and consequently expression of ATGs is the result of decreased PGC1α levels and/or PGC1α activity in melanomas. More accessible melanoma samples, preferably analyzed with latest functional genomics methods would be needed to further investigate NRF1 binding.

On the other hand, silencing of NRF1 could, in addition to the expression of ATG5 and ATG7, target several other genes, which could positively or negatively influence the migration potential of melanoma cells. Therefore, the impact NRF1 on the migration could also be autophagy-independent. Indeed, knockdown of NFE2L2 decreased the expression of ATGs but had no impact on the migration potential of melanoma cells. In addition, several of proteins regulating autophagy, including ATG5 and ATG7, have been shown to exhibit autophagy-independent functions (41). Taken together, the migration of melanoma cells might be regulated by ATG5 and ATG7 independent on their role in autophagy.

Currently, there is an immense interest in the therapeutic modulation of autophagy, which has to be tightly regulated, since both excess and lack of autophagy can be damaging to the cell. Thus, it is crucial to study the mechanisms that control autophagy in order to ensure safe therapeutic regulation in several diseases, including cancer. In addition to our previous study where we demonstrated decreased expression of Beclin-1 in metastatic melanomas (8), we show here that the expression of two more ATG proteins, ATG5 and ATG7, are reduced in primary and metastatic melanomas compared to benign nevi. Furthermore, the combination of LC3B and p62 expression analysis revealed that autophagy is likely to be reduced in melanoma tissues. Importantly, downregulation of both ATG5 and ATG7 correlates with a less favourable outcome for patients, suggesting the potential use of ATG5 and ATG7 levels as prognostic markers in melanoma in order to recognize the more aggressive, metastatic phenotype. Furthermore, we identified NRF1 as a new transcription factor involved in the regulation of ATG5 and ATG7, improving our current understanding of the regulation of autophagy during melanoma development and progression.

## Data Availability Statement

The original contributions presented in the study are included in the article/**Supplementary Material**. Further inquiries can be directed to the corresponding author.

## Ethics Statement

The studies involving human participants were reviewed and approved by Ethics Committee of the Canton of Bern. Written informed consent for participation was not required for this study in accordance with the national legislation and the institutional requirements.

## Author Contributions

ŽF planned and performed the study, analysed and interpreted data and wrote the manuscript. DG and LS performed the TEPIC analysis. PL performed the GSE3189 analysis. ZH performed the quantification of the TMA. MG performed experimental analysis. SMSJ and YF-M performed experiments. REH took clinical care of the melanoma patients. TS gave advice on bioinformatic analysis and provided laboratory infrastructure. SY supervised and provided experimental advice. LS supervised and edited the manuscript. H-US supervised, provided experimental advice and laboratory infrastructure, and edited the manuscript. All authors contributed to the article and approved the submitted version.

## Funding

This work was supported by the Swiss National Science Foundation (grant number 310030_184816) and European Union Horizon 2020 Research and Innovation Program (Marie Sklodowska-Curie grant No. 642295; MEL-PLEX), and Russian Government Program “Recruitment of the Leading Scientists into the Russian Institutions of Higher Education”, grant No. 075-15-2021-600 (H-US).

## Conflict of Interest

The authors declare that the research was conducted in the absence of any commercial or financial relationships that could be construed as a potential conflict of interest.

## Publisher’s Note

All claims expressed in this article are solely those of the authors and do not necessarily represent those of their affiliated organizations, or those of the publisher, the editors and the reviewers. Any product that may be evaluated in this article, or claim that may be made by its manufacturer, is not guaranteed or endorsed by the publisher.
