# Corrigendum: ATG5 and ATG7 expression levels are reduced in cutaneous melanoma and regulated by NRF1

**DOI:** 10.3389/fonc.2025.1549776

**Published:** 2025-01-17

**Authors:** Živa Frangež, Deborah Gérard, Zhaoyue He, Marios Gavriil, Yuniel Fernández-Marrero, S. Morteza Seyed Jafari, Robert E. Hunger, Philippe Lucarelli, Shida Yousefi, Thomas Sauter, Lasse Sinkkonen, Hans-Uwe Simon

**Affiliations:** ^1^ Institute of Pharmacology, University of Bern, Bern, Switzerland; ^2^ Department of Life Sciences and Medicine, University of Luxembourg, Belvaux, Luxembourg; ^3^ Biological Sciences Platform, Sunnybrook Research Institute, Sunnybrook Health Science Centre, Toronto, ON, Canada; ^4^ Department of Dermatology, Inselspital, Bern University Hospital, University of Bern, Bern, Switzerland; ^5^ Institute of Biochemistry, Medical School Brandenburg, Neuruppin, Germany; ^6^ Department of Clinical Immunology and Allergology, Sechenov University, Moscow, Russia; ^7^ Laboratory of Molecular Immunology, Institute of Fundamental Medicine and Biology, Kazan Federal University, Kazan, Russia

**Keywords:** autophagy, ATG5, ATG7, melanoma, NRF1, transcription factor

In the published article, there was an error in **Figure 6D** as published. During the final preparation of the figures, an incorrect image was inadvertently included in **Figure 6D**, specifically the representation of the Nevus tissue. This error occurred during the process copy and pasting images into the PowerPoint file used for the figures. We would like to emphasize that this mistake does not affect the scientific integrity of the paper. The quantitative data on NRF1 expression, as shown in the left panel of **Figure 6D**, remains accurate and unaffected by the incorrect image. We have since corrected **Figure 6D** by replacing the erroneous images with the correct representative images. The corrected **Figure 6D** and its caption appear below.

**Figure 6:**
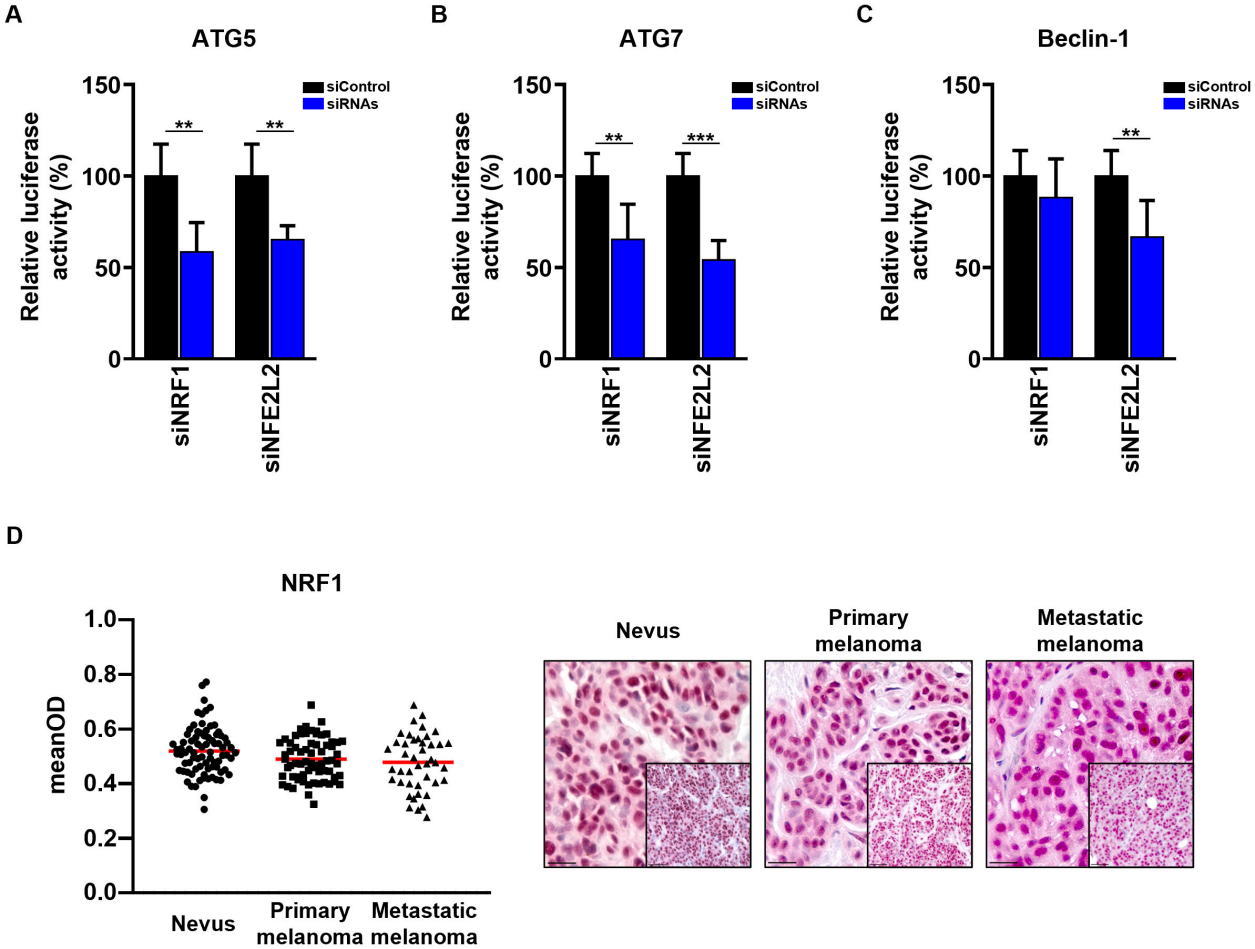
Validation of NRF1 and NFE2L2 regulation of ATG5, ATG7 and Beclin-1 promoter regions. **(A–C)** The Dual-Luciferase reporter plasmids (pGL4.74 [hRluc/TK] and pGL4.15[luc2P/Hygro]) were co-transfected with 50 nM siRNAs (Dharmacon) into SK-MEL-5 melanoma cells. 48 h post transfection cells were assayed for luciferase activity. The firefly luciferase activities were normalized to renilla luciferase activity. The firefly luciferase activity of the cells transfected with siRNAs is represented as the percentage of activity relative to that of the cells transfected with Non-Targeting siRNA (siControl). Statistical differences were analyzed by multiple t-test using the Holm-Sidak correction method (n=3). p ≤ 0.01**; p ≤ 0.001***. **(D)** Immunohistochemistry. Quantification of the NRF1 signal intensity in the nucleus. Intensity (mean optical density (meanOD)) values for individual patients are presented. The red lines represent the mean of all values. Statistical differences were analyzed by one-way ANOVA using a Kruskal-Wallis test and Dunn’s *post hoc* test (left panel). Representative images of 80 benign nevi as well as 67 primary and 43 metastatic melanomas are shown (right panel). Scale bars 20 μm (inlets) and 50 μm.

The authors apologize for this error and state that this does not change the scientific conclusions of the article in any way. The original article has been updated.

