# Supplementary figures and images for: ATG5 and ATG7 Expression Levels Are Reduced in Cutaneous Melanoma and Regulated by NRF1
Source: Front Oncol. 2021 Aug 12;11:721624. doi: 10.3389/fonc.2021.721624 (PMC8397460; doi:10.3389/fonc.2021.721624)

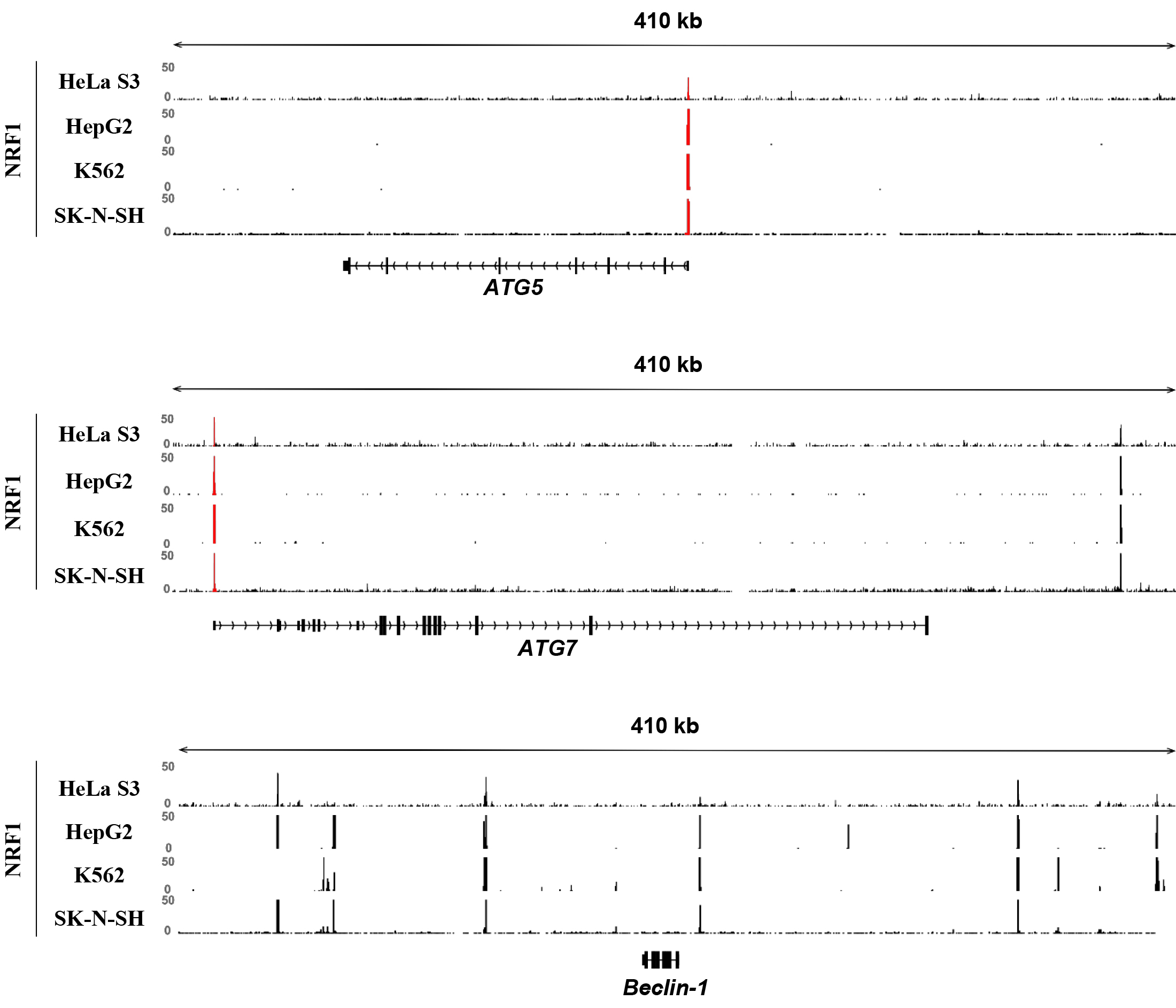

Supplement: Supplementary file 1 [file Image_1.jpg]

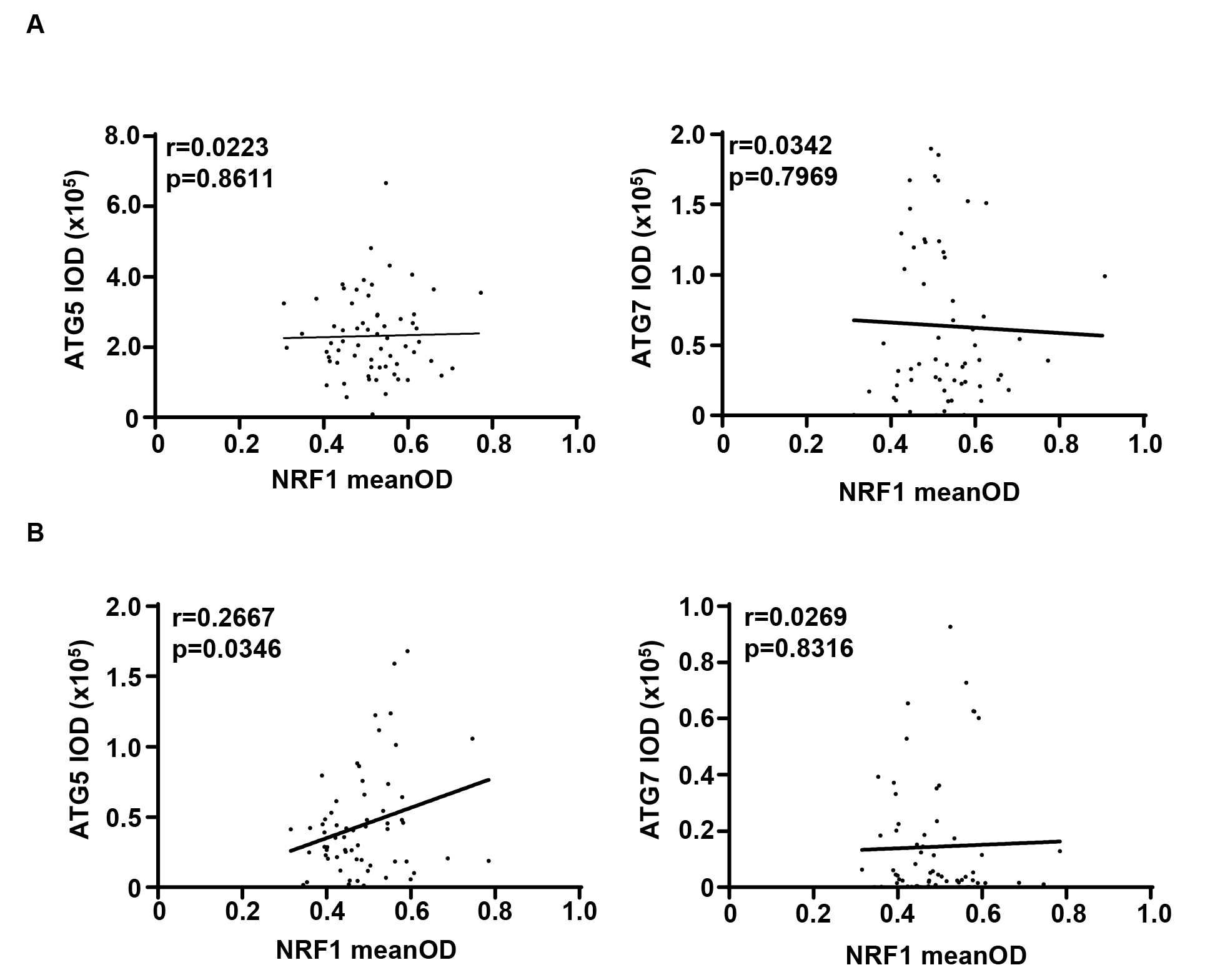

Supplement: Supplementary file 2 [file Image_2.jpg]
