# Supplementary material for: ATG5 and ATG7 Expression Levels Are Reduced in Cutaneous Melanoma and Regulated by NRF1
Source: Front Oncol. 2021 Aug 12;11:721624. doi: 10.3389/fonc.2021.721624 (PMC8397460; doi:10.3389/fonc.2021.721624)
Supplement: Supplementary file 3 [file Table_1.docx]

Table S1: Top 10 motifs with highest affinity scores for *ATG5* promoter region

| Chromosome | Start | End | Peaks | Transcription factors (TFs) | TFs affinities |
| --- | --- | --- | --- | --- | --- |
| 6 | 106325764 | 106325860 | 6:106325763-106325860 | MXI1 | 0.895598 |
| 6 | 106325764 | 106325860 | 6:106325763-106325860 | CREB1 | 0.774078 |
| 6 | 106325764 | 106325860 | 6:106325763-106325860 | MYCN | 0.524294 |
| 6 | 106325764 | 106325860 | 6:106325763-106325860 | MAX | 0.42564 |
| 6 | 106325764 | 106325860 | 6:106325763-106325860 | MYC | 0.324955 |
| 6 | 106325764 | 106325860 | 6:106325763-106325860 | JUN | 0.204571 |
| 6 | 106325764 | 106325860 | 6:106325763-106325860 | MNT | 0.179946 |
| 6 | 106325764 | 106325860 | 6:106325763-106325860 | ATF7 | 0.143572 |
| 6 | 106325764 | 106325860 | 6:106325763-106325860 | NFIX | 0.112731 |
| 6 | 106325764 | 106325860 | 6:106325763-106325860 | TFEC | 0.096109 |
