# Supplementary material for: ATG5 and ATG7 Expression Levels Are Reduced in Cutaneous Melanoma and Regulated by NRF1
Source: Front Oncol. 2021 Aug 12;11:721624. doi: 10.3389/fonc.2021.721624 (PMC8397460; doi:10.3389/fonc.2021.721624)
Supplement: Supplementary file 4 [file Table_2.docx]

Table S2: Top 10 motifs with highest affinity scores for *ATG7* promoter region

| Chromosome | Start | End | Peaks | Transcription factors (TFs) | TFs affinities |
| --- | --- | --- | --- | --- | --- |
| 3 | 11272287 | 11272376 | 3:11272286-11272376 | NRF1 | 0.506994 |
| 3 | 11272287 | 11272376 | 3:11272286-11272376 | RBPJ | 0.153636 |
| 3 | 11272287 | 11272376 | 3:11272286-11272376 | MZF1 | 0.100512 |
| 3 | 11272287 | 11272376 | 3:11272286-11272376 | THAP1 | 0.073209 |
| 3 | 11272287 | 11272376 | 3:11272286-11272376 | HIC2 | 0.020404 |
| 3 | 11272287 | 11272376 | 3:11272286-11272376 | NFIX | 0.020005 |
| 3 | 11272287 | 11272376 | 3:11272286-11272376 | HIF1A | 0.016132 |
| 3 | 11272287 | 11272376 | 3:11272286-11272376 | RELB | 0.012664 |
| 3 | 11272287 | 11272376 | 3:11272286-11272376 | REL | 0.009337 |
| 3 | 11272287 | 11272376 | 3:11272286-11272376 | TEAD3 | 0.009283 |
